# Combination of Oxyanion Gln114 Mutation and Medium Engineering to Influence the Enantioselectivity of Thermophilic Lipase from *Geobacillus zalihae*

**DOI:** 10.3390/ijms130911666

**Published:** 2012-09-17

**Authors:** Roswanira Abdul Wahab, Mahiran Basri, Mohd Basyaruddin Abdul Rahman, Raja Noor Zaliha Raja Abdul Rahman, Abu Bakar Salleh, Thean Chor Leow

**Affiliations:** 1 Enzyme and Microbial Technology Group, Faculty of Science, Universiti Putra Malaysia, Serdang, Selangor 43400, Malaysia; E-Mail: basya@science.upm.edu.my; 2 Faculty of Science, Universiti Teknologi Malaysia, Skudai, Johor 81310, Malaysia; 3 Enzyme and Microbial Technology Group, Faculty of Biotechnology and Biomolecular Sciences, Universiti Putra Malaysia, Serdang, Selangor 43400, Malaysia; E-Mails: rnzaliha@biotech.upm.edu.my (R.N.Z.R.A.R.); abubakar@biotech.upm.edu.my (A.B.S.); adamleow@biotech.upm.edu.my (T.C.L.); 4 Institute of Bioscience, Universiti Putra Malaysia, UPM Serdang, Selangor 43400, Malaysia

**Keywords:** enantioselectivity, T1 lipase, organic solvents, *Geobacillus zalihae*, site-directed mutagenesis, desiccant

## Abstract

The substitution of the oxyanion Q114 with Met and Leu was carried out to investigate the role of Q114 in imparting enantioselectivity on T1 lipase. The mutation improved enantioselectivity in Q114M over the wild-type, while enantioselectivity in Q114L was reduced. The enantioselectivity of the thermophilic lipases, T1, Q114L and Q114M correlated better with log *p* as compared to the dielectric constant and dipole moment of the solvents. Enzyme activity was good in solvents with log *p* < 3.5, with the exception of hexane which deviated substantially. Isooctane was found to be the best solvent for the esterification of (*R*,*S*)-ibuprofen with oleyl alcohol for lipases Q114M and Q114L, to afford *E* values of 53.7 and 12.2, respectively. Selectivity of T1 was highest in tetradecane with *E* value 49.2. Solvents with low log *p* reduced overall lipase activity and dimethyl sulfoxide (DMSO) completely inhibited the lipases. Ester conversions, however, were still low. Molecular sieves employed as desiccant were found to adversely affect catalysis in the lipase variants, particularly in Q114M. The higher desiccant loading also increased viscosity in the reaction and further reduced the efficiency of the lipase-catalyzed esterifications.

## 1. Introduction

Genetic engineering, through significant advances in site-directed mutagenesis and directed evolution, is a series of strategies available to modify and control almost any enzyme property [1]. Both strategies can endow enzymes with super ability against high temperatures, organic reagents and denaturants [2] and are extensively used to enhance enzyme activity and enantioselectivity [1]. Enantioselectivity can be genetically engineered into existing enzymes by isolating novel biocatalysts or by engineering the medium of reaction [3]. Changing the solvents employed in the reactions is one method in medium engineering that can alter enantioselectivity [4–6]. However, there is still no general understanding between solvent physicochemical properties and enzyme enantioselectivity due to the diversity of substrates and enzymes used [7].

Thermophilic enzymes are more thermally active and stable in organic solvents than other enzymes [8] and have significant potential applications in biotechnological processes [9]. Previously, we described a thermoalkalophilic lipase, called T1, from *Geobacillus zalihae* gene encoding 388 aa residues using vector pGEX 4-T1, that was highly expressed in recombinant *E. coli* BL21 (DE3) pLys*S* [10]. The catalytic machinery is formed by Ser-114, His-359 and Asp-314 [11]. The oxyanion of T1 lipase was deduced to be Q114 and F16, based on the BTL2 lipase oxyanion at Q115 and F17 that shares 96% sequence similarity to T1 lipase. The F17 residue in BTL2 lipase was reportedly to be highly conserved [12] and the same was expected of the F16 in T1 lipase. However, substitution at Q114 was anticipated to be less damaging and more tolerable.

It has been described that mutations near the enzyme active site can enhance enantioselectivity more strongly than distant mutations [13]. The effect of mutating the oxyanion Q114 on enantioselectivity of T1 lipase has yet to be explored. We substituted the hydrophilic Q114 with hydrophobic Leu and Met, to afford lipase variants Q114L and Q114M. Both lipase variants and the wild-type were then compared in the enantioselective esterification of ibuprofen in mixtures that consisted of different solvents and variable desiccant loading. The intention of this article was to show that the oxyanion at site 114 plays a role in imparting and controlling enantioselectivity in T1 lipase. The influence of different properties of solvents and variable amounts of desiccant were also examined in order to obtain a better perception on how both parameters affected enantioselectivity.

## 2. Results and Discussion

### 2.1. Effect of Solvents

**Scheme 1:**
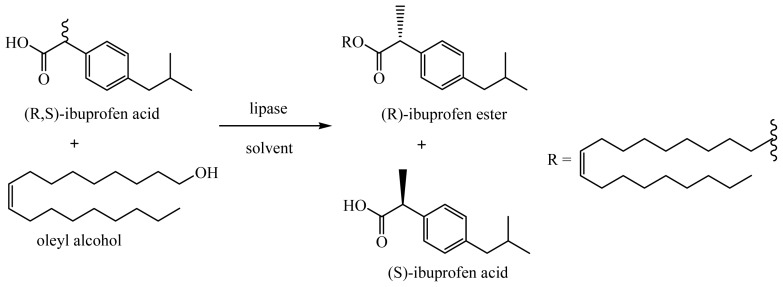
The reaction scheme for the esterification of (*R*,*S*)-ibuprofen with oleyl alcohol catalyzed by lipase variants T1, Q114L and Q114M in solvent.

In the resolution of racemic ibuprofen, oleyl alcohol was used as the resolving agent to separate the enantiomers of ibuprofen (Scheme 1). Recent molecular dynamic simulations revealed that the activation mechanism of T1 lipase involved the lid domain. It is formed by the helix-loop-helix motif that was proposed to be involved in interfacial activation of T1 lipase. The large structural rearrangement of the lid that reveals the entrance to the active site only occurs as a result of interaction between the hydrophobic residues of the lid with octane [14]. Hence, oleyl alcohol was chosen, as it a natural substrate that T1 lipase normally catalyzes and the hydrophobicity of the oleyl alcohol is pertinent for the activation of catalysis.

**Figure 1:**
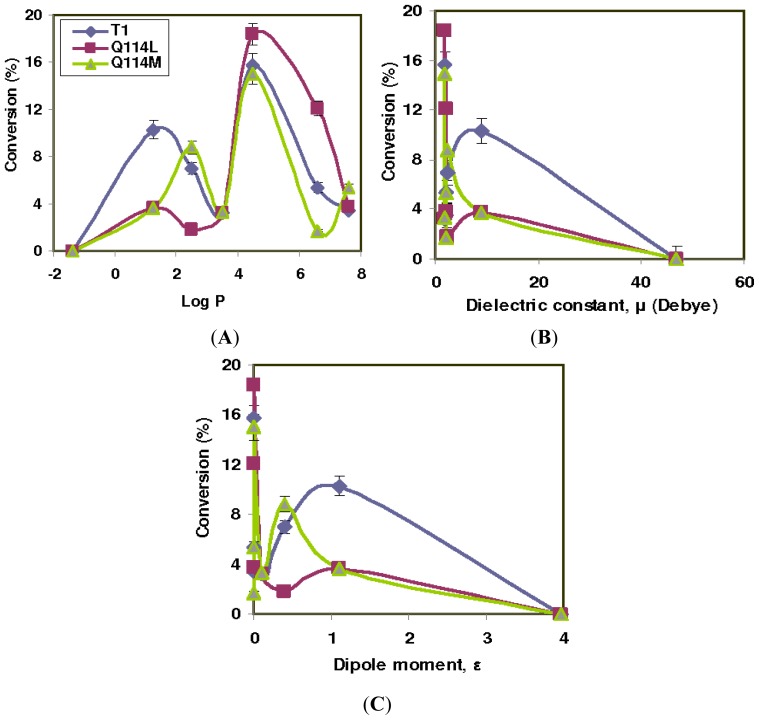
The effects of various solvent properties on percentage conversion in the T1 lipase, Q114L and Q114M-catalyzed esterification of ibuprofen. The reactions of the lipase variants were carried out under similar conditions in solvents, dimethyl sulfoxide (DMSO), acetonitrile, ethyl acetate, hexane, isooctane, toluene, dodecane and *N*-tetradecane (8 mL), lyophilized lipase (5 mg) (*R*,*S*)-ibuprofen (0.8252 g, 4 mmole) and oleyl alcohol (1.872 mL, 6 mmole) at 50 °C and stirred at 200 rpm. The solvent properties investigated were (**A**) log *p*; (**B**) dielectric constant and (**C**) dipole moment.

The effect of different solvent properties on the esterification of racemic ibuprofen with oleyl alcohol catalyzed by T1, Q114L and Q114M were evaluated and the results are illustrated in Figure 1. The lipase activity in the esterification reaction was represented by the conversion of the ibuprofen ester. Activity in reactions of T1, Q114L and Q114M were found to be good when log *p* of solvents were 1.25 ≤ log *p* ≤ 7.6, but were inactivated in dimethyl sulfoxide (DMSO) (log *p* = −1.23). Their activity was noted when solvent dipole moment, *μ* < 10 or dielectric constant, *ɛ* < 0.1. The highest conversion catalyzed by T1, Q114L and Q114M occurred in isooctane which corresponded to 15.7%, 18.4% and 15.0%, respectively.

The activity of the lipase variants improved with decreasing solvent polarity (higher log *p*), except in hexane. The activity of the lipase variants declined significantly when in hexane. For T1 lipase, the lowest conversion occurred in hexane at 3.2%, Q114L in toluene at 1.8% and Q114M in dodecane at only 1.7%. DMSO completely inhibited activity in all lipase variants. The lipase variants exhibited low conversions (<5%) in dichloromethane and *N*-tetradecane. Only T1 lipase showed good activity in dichloromethane with ester conversion at 10.3%. It was noted that ester conversions in T1, Q114L and Q114M-catalyzed reactions correlated better with the log *p* of solvents.

**Figure 2:**
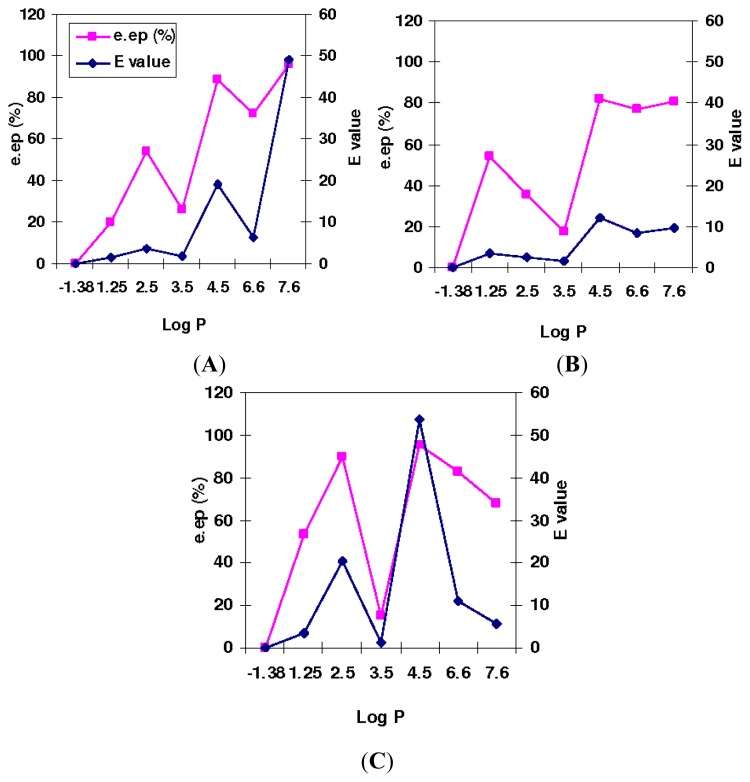
The effects of solvent log *p* on e.e*_p_*% and *E* value in the T1 lipase, Q114L and Q114M-catalyzed esterification of ibuprofen. The reactions were carried out in solvents, DMSO, acetonitrile, ethyl acetate, hexane, isooctane, toluene, dodecane and *N*-tetradecane (8 mL), lyophilized lipase (5 mg) (*R*,*S*)-ibuprofen (0.8252 g, 4 mmole) and oleyl alcohol (1.872 mL, 6 mmole) at 50 °C and stirred at 200 rpm. (**A**) T1; (**B**) Q114L and (**C**) Q114M.

The *E* values and e.e*_p_*% in T1, Q114L and Q114M-catalyzed enantioselective esterification of ibuprofen with respect to solvent log *p* is illustrated in Figure 2. Generally, e.e*_p_*% increased with increasing log *p* with notable common deviation when in hexane, whereby e.e*_p_*% of all lipase-catalyzed reactions significantly dropped. T1 also showed slightly reduced e.e_p_% in dodecane and in reactions of Q114M, e.e*_p_*% was considerably reduced when log *p* exceeded 4.5. Meanwhile, *E* values of reactions varied between 1.4 to 53.7 and were particularly poor at low solvent log *p*. Conversely, *E* values gradually improved when log *p* of solvents were increased, with the exception of hexane.

Similar deviations were also observed for reactions of T1 and Q114L in dodecane. Good selectivity was achieved when dielectric constants and dipole moments were lowest and selectivity of the lipase variants almost diminished when in hexane (*E* < 2). However, due to poor correlation between ester conversion with solvent dipole moment and dielectric constant, the same outcome was also expected of *E* values in the lipase-catalyzed reactions.

Selectivity of T1 lipase improved significantly in isooctane and *N*-tetradecane to give E value 19.1 in isooctane, and its best *E* value at 49.2 in *N*-tetradecane. *E* values for Q114L were 12.2 and 9.7 in isooctane and *N*-tetradecane, respectively. Selectivity of Q114M was highest in isooctane to give E value 53.7 and a moderate *E* value 20.5 in dichloromethane. Unlike T1 and Q114L, Q114M performed poorly in *N*-tetradecane with *E* value 5.5, as with T1 lipase in dichloromethane. *E* values of T1 lipase also deviated slightly in dodecane with a sharp decline in *E* value at 6.4, followed by a sharp increase in *E* value to 49.2 in *N*-tetradecane (log *p* 7.6).

It is well reported in literature that enzyme activity and selectivity are strongly affected by the choice of organic solvents [15–18]. In addition, using organic solvents in reactions can also provide the advantage of shifting the thermodynamic equilibria to favor synthesis over hydrolysis [18]. It was observed that all three lipases were completely inhibited in DMSO, which can be ascribed to the high polarity of DMSO as opposed to the other solvents that were used. Our finding corroborated other reports that highly polar solvents were responsible for the stripping of the essential water from the protein and disrupting the structure of the enzyme [19].

Hydrophilic solvents, such as DMSO, used in the reactions have been described as having higher affinity towards water rather than to the enzyme. As a consequence, there was a loss of conformational flexibility in the enzyme due to lack of bound water [15,20,21], which resulted in the loss of activity as well as enantioselectivity. Also, these solvents have been known to induce changes in resonances of amide bonds of surface amino acids in enzymes, as previously reported for *Candida antarctica* lipase B when in the presence of acetonitrile [22]. This would have certainly caused intermittent adoption of unreactive enzyme conformation and temporary loss of structural rigidity. Resonances of the amide bonds meant active fluctuations between single bond and double character on the carbonyl group, making the enzyme surface highly unstable, comparable to a molten globule-like intermediate structure [23]. In essence, hydrophilic solvents impede enzyme activity, and not just because they strip water from the enzyme [24]. On the other hand, the high ester conversions in isooctane suggested that it was a good solvent for the lipase-catalyzed esterification. This finding was similar to a report that illustrated that esterification of ketoprofen by *Candida rugosa* afforded good results when cyclohexane or isooctane was used as the main solvent [25]. In another study, the subtilisin lipase retained its active native environment, including its structural water upon treatment with octane (log *p* 4.9). They discovered that the bulk of the solvent partitioned away from the active site of the lipase [26]. A similar phenomenon may have occurred for Q114M in isooctane (log *p* 4.5), which may account for its more efficient catalysis during the esterification reaction. Also, the comparatively more hydrophobic methionine residue in Q114M would not have displaced water due to its opposing hydrophobicity.

Meanwhile, the particularly low ester conversion in dichloromethane can be attributed to the increased difficulty of substrate accessibility into the active site. The catalytic pocket of lipase is largely available to external solvents and substrates through a rather narrow hydrophobic channel [27]. For lipases, T1, Q114L and Q114M, the hydrophobic lid was the additional obstacle before the catalytic pocket could be accessed. The lid tends to interact more strongly with hydrophobic solvents in order for it lid to open. Hence, the use of less hydrophobic solvents resulted in a weaker activation of the hydrophobic lid and increased substrate difficulty to access the active site. Hence, the use of less hydrophobic solvents could have resulted in a weaker activation of the hydrophobic lid, given the fact that the lid domain of T1 lipase is involved in interfacial activation [14]. Similar observations were also reported for an esterification reaction catalyzed by the CALB enzyme [28]. This might partially explain the observed poor conversion of ibuprofen ester for T1, Q114L and Q114M-catalyzed reactions in dichloromethane. Furthermore, it has been described in literature that solvents can also influence the ground state of the reactants and products. A higher intrinsic solubility of the substrates in organic solvents tends to thermodynamically stabilize the substrate ground state and decrease enzyme activity [26].

The *E* values of the thermophilic lipase variants, T1 lipase, Q114L and Q114M correlated better with hydrophobicity of the solvent rather than the dielectric constant and dipole moment. Results from the solvent properties, dielectric constant and dipole moment were less conclusive due to poor association between the *E* values and both solvent properties. Good correlation between the *E* values and solvent log *p* were in accordance with reports that linked enantioselectivity of enzymatic reaction with hydrophobicity of solvent [29] and could be used as a guide to estimate the solvent effects in T1, Q114L and Q114M-catalyzed esterification of racemic ibuprofen with oleyl alcohol. Only one of the solvents investigated, namely hexane, deviated substantially in this respect. Another noteworthy point to be considered was the solubility of the substrate and product in the various solvents. The poor solubility of ibuprofen in some organic solvents has been known to decrease enzyme activity and result in a lower conversion and E value [30].

### 2.2. Effect of the Presence of Desiccant

**Figure 3:**
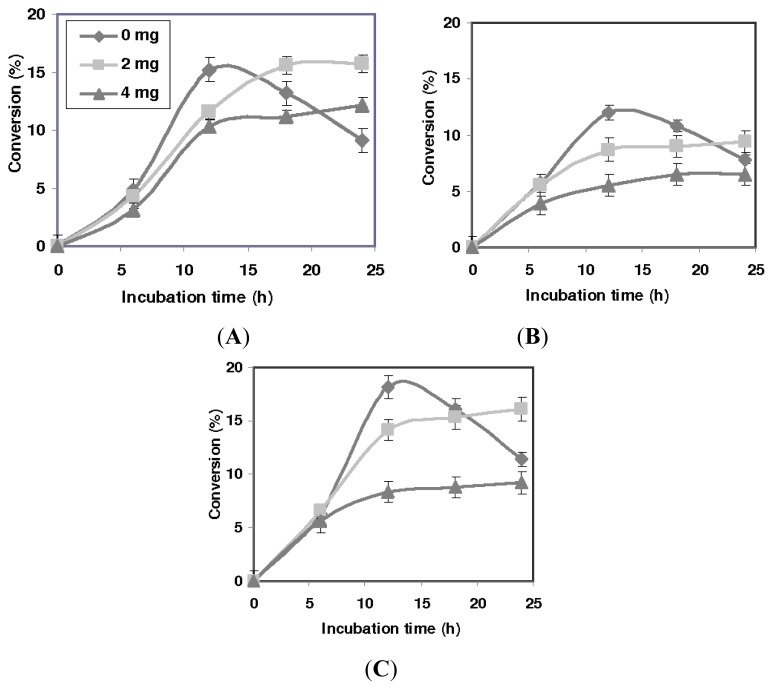
The time course of the T1 lipase, Q114L and Q114M-catalyzed esterification of ibuprofen. The reaction was carried out under similar conditions, in isooctane (8 mL), lyophilized lipase (5 mg), (*R*,*S*)-ibuprofen (0.8252g, 4 mmole) and oleyl alcohol (1.872 mL, 6 mmole) at 50 °C and stirred at 200 rpm. (**A**) T1; (**B**) Q114L and (**C**) Q114M.

The effect of variable desiccant loading on the activity of the lipase variants is represented as ester conversion. The results for the T1, Q114L and Q114M-catalyzed esterification of racemic ibuprofen are shown in Figure 3. The molecular sieves quickly dispersed after a few minutes of stirring and caused the medium to turn slightly viscous, particularly in the medium of the 4 mg loading.

In comparison, lipase activity was higher in reactions without added molecular sieves, as exemplified in the higher ester conversions. However, in the absence of molecular sieves, ester conversion started to decline when the incubation period exceeded 12 h. The T1, Q114L and Q114M variants afforded their highest conversion at 12 h, corresponding to 15.2%, 12.0% and 18.1%, respectively. Conversely, mixtures containing desiccant achieved equilibrium at a much later time. In the 2 mg loading, T1 and Q114L achieved equilibrium after 18 h, but conversion was still rising in the Q114M-catalyzed reaction. At 4 mg loading, only T1 and Q114M showed marginal increase in conversion. However, Q114L attained equilibrium much sooner, at approximately 18 h, with considerably lower enzyme activity as compared to T1 and Q114M.

**Figure 4:**
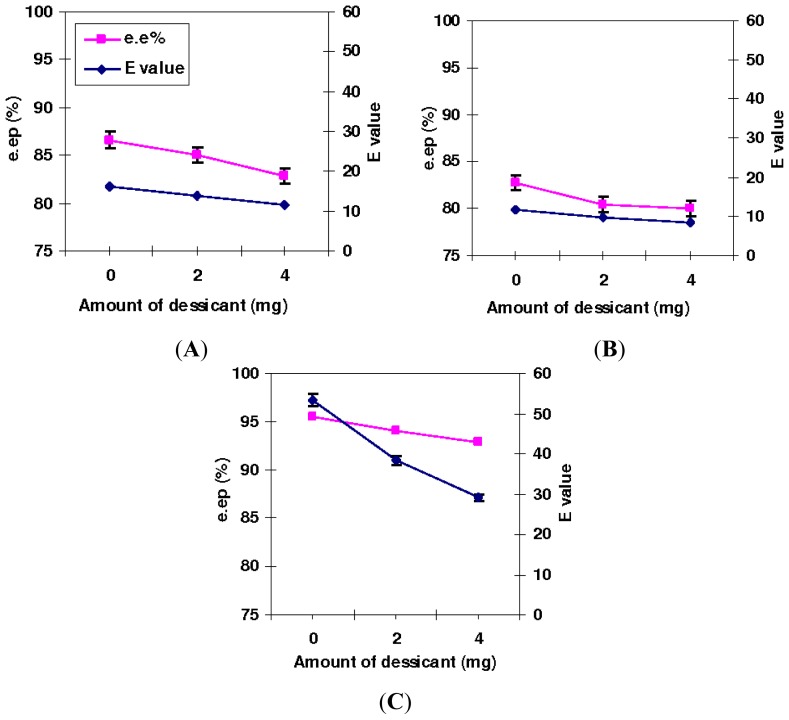
The effects of desiccant loading on e.e*_p_*% and *E* values in the T1 lipase, Q114L and Q114-M catalyzed esterification of ibuprofen. The reactions were carried out in isooctane (8 mL), lyophilized lipase (5 mg) (*R*,*S*)-ibuprofen (0.8252 g, 4 mmole) and oleyl alcohol (1.872 mL, 6 mmole) at 50 °C and stirred at 200 rpm. The amount of added desiccant was examined at 0, 2 and 4 mg loading. (**A**) T1; (**B**) Q114L and (**C**) Q114M.

The *E* values and e.e*_p_*% of T1, Q114L and Q114M-catalyzed esterification with respect to the amount of desiccant loading are illustrated in Figure 4. In all reactions catalyzed by the three lipases, e.e*_p_*% reduced as desiccant loading was increased. Selectivity was highest for reactions without added desiccant with Q114M exhibiting the highest *E* values—between 29.1 and 53.4. Selectivity profiles for T1 and Q114L under variable desiccant loading were quite similar, except selectivity, which was marginally higher in the former at *E* values 11.7 to 13.8, while the latter attained *E* values 8.6 to 11.8.

It was clear that the enantioselectivity of the lipase variants was particularly low in reactions that contained molecular sieves as desiccant. The effect of the desiccant on the reactions, however, was more profound in the Q114M variant. The *E* values of Q114M were significantly reduced from 53.4 to 29.1 when the desiccant loading was increased from 0 mg to 4 mg. However, *E* values of T1 and Q114L lipase were less affected, although the enzymes were considerably less selective than Q114M, regardless of the amount of additives that were used. Q114L was found to be the least selective and the *E* values in the T1 and Q114L-catalyzed reactions declined gradually over time. It is a well-known fact that an esterification reaction releases water as a by-product, which favors hydrolysis and counteracts esterification. The long incubation time of T1, Q114L and Q114M-catalyzed reactions at high temperatures would have increased water buildup. This could have considerably affected reaction equilibrium and influence the e.e% of product or unreacted substrate. To overcome this, displacement of the equilibrium towards formation of product using additives would certainly improve enantiomeric excess, particularly when the enzyme has low or moderate enantioselectivity [8]. In this study, direct addition of desiccant into the reaction medium was favored, as it was by far a much simpler method to remove generated water.

Using molecular sieves as desiccant to remove water generated from the reaction was reportedly an effective way to increase conversion of substrates in an esterification reaction. However, the adverse effects of the molecular sieves on the lipases, especially in the Q114M-catalyzed reactions, implied that there could be some interaction between the lipase and molecular sieves. The stirring further intensified lipase-molecular sieve interaction that damaged the protein structures and reduced activity. Moreover, high loading of the additives would have added more bulk to the reaction. This caused low collision probability between enzyme and substrate, which further reduced the efficiency of catalysis [20]. It can be concluded that addition of molecular sieves to remove generated water in the reactions was not suitable and use of an alternative desiccant should be considered.

### 2.3. The Effect of Mutation on Enantioselectivity

**Figure 5:**
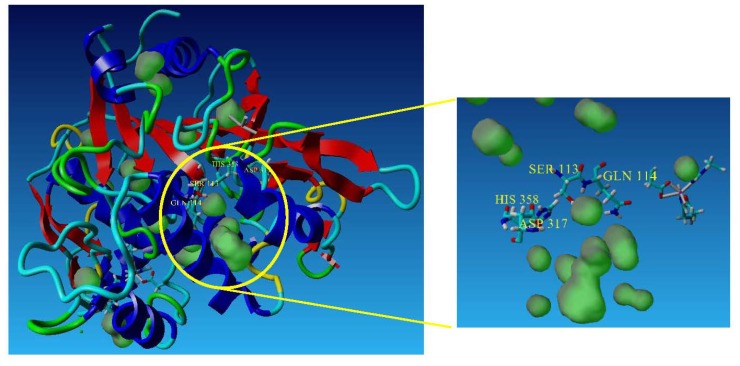
The location of the oxyanion Gln114 that was selected for site-directed mutagenesis in the catalytic pocket of T1 lipase with respect to the catalytic triad, Ser113, His358 and Asp317.

The oxyanion Q114 in T1 lipase selected for site-directed mutagenesis is located next to the catalytic Ser, and is important in the stabilization of the substrate intermediate during catalysis (Figure 5). Mutations near the active site have been proposed to enhance enantioselectivity more strongly than distant mutations [13]. The differences in enantioselectivities between the lipase variants could have been attributed to the more hydrophobic Leu and Met residues at the as compared to the hydrophilic Gln in T1 lipase. In terms of hydrophobicity, Met (Q114M) is more hydrophobic, followed by Leu (Q114L), and finally Gln (T1 lipase). According to reports, high enantioselectivity in enzymes have been associated with conformational rigidity of enzyme structure [31]. Active sites that are more rigid will result in an enzyme that is more enantioselective, due to formation of a more rigid binding pocket that can permit only one enantiomer to properly enter the active site [32]. With regards to this, Met being the most hydrophobic was expected to reduce the flexibility in the binding pocket in Q114M: hence, enhancing its rigidity. Thus, it might explain the observed moderate improvement in enantioselectivity of the lipase. The proponent that enhanced enantioselectivity in Q114M could be the increased rigidity in its active site. In the case of Q114L, substitution with the hydrophobic Leu clearly did not improve selectivity of the enzyme. Apart from modulation of the rigidity in the binding pocket, replacement of Gln in T1 lipase could have also invoked other substantial changes, such as altering the shape and/or size of the active site [33]. Substitution of the bulky Gln residue with a much smaller Leu residue would logically free up more space at the substrate binding site and alleviate steric hindrance, allowing entrance of both enantiomers of ibuprofen acid. Hence, arrangements of residues in the vicinity of the mutation are disrupted, altering active site topology of Q114L that compromised enantiorecognition or chirality in the active site of the lipase.

## 3. Experimental Section

### 3.1. Materials

The components for the growth media were procured from Difco Laboratories (USA). The culture harboring the recombinant T1 lipase gene was obtained from stock cultures from the Enzyme and Microbial Technology Laboratory, UPM. The BL21 (DE3) pLys*S* cultures were from Invitrogen (Groningen, The Netherlands). The Quick-Change™ Site-Directed Mutagenesis Kit was purchased from Stratagene (La Jolla, CA, USA). Agar plates were prepared by addition of tributyrin to Luria-Bertani (LB) agar (Oxoid, UK) and antibiotics, chloramphenicol (35 mg/mL) and ampicillin (50 mg/mL) were supplied by Amresco (Solon, Ohio, USA). The Bradford reagent was also from Amresco (Solon,Ohio, USA). Oleyl alcohol was purchased from Fluka (Buchs, Switzerland). Ibuprofen and molecular sieves 3 Ǻ were purchased from Sigma-Aldrich (St. Louis, MO, USA). The solvents, DMSO, acetonitrile, ethyl acetate, hexane, toluene, isooctane, dodecane and *N*-tetradecane, were of HPLC grade and were obtained from Merck (Darmstadt, Germany). Deionized water was produced in our laboratory.

### 3.2. Substitution of Residues by Site-Directed Mutagenesis

The Q114L and Q114M lipase proteins were made using the QuickChange method. The PCR reaction mixture was set up according to manufacturer’s protocol. The primers were designed with the sequence change located in the center of the primer with approximately 10–15 bases on both sides. The DNA was incubated with the restriction enzyme D*pn-*1 at 37 °C for 1 h to digest the parental methylated DNA. The D*pn-*1-digested DNA was introduced into competent *E. coli* BL21 (DE3) pLys*S* cells, and the cultures were grown on LB-tributyrin agar media containing ampicillin and chloramphenicol. Each mutation was confirmed by sequencing. Glycerol stocks were then prepared and kept at −80 °C.

### 3.3. Preparation of Stock and Working Culture

The stock culture was prepared by inoculating a colony of lipase-producing bacteria into aliquots of Luria Bertani (LB) broth and incubated for 12–16 h. Aliquots of LB broth (800 μL) were added to sterile 15% glycerol (200 μL) in 1.5 mL Eppendorf tubes, vortexed and stored at −80 °C. As for the working culture, one loop of culture from the glycerol stock was streaked onto tributyrin-nutrient agar-ampicillin plates and incubated for 12–16 h at 37 °C. A carefully selected single colony was inoculated aseptically into a 10 mL sterile LB broth and incubated for 12–16 h at 37 °C and shaken at 200 rpm. The bacterial culture was centrifuged at 10,000 rpm for 10 min and stored at −80°C.

### 3.4. Partial Purification of Enzymes

The recombinant culture (1000 mL) was harvested by centrifugation and resuspended with 40 mL of PBS (pH 7.4) containing 5 mM of DTT prior to sonication. The cell lysate was cleared by centrifugation at 12,000× *g* for 30 min and filtered with 0.45 μm membrane filter (Sartorius). The resin glutathione-sepharose HP (10 mL) was packed into an XK 16/20 column (GE Healthcare) and was with ten-column volume (CV) of PBS (pH 7.4). The cleared cell lysate was loaded on a Glutathione-Sepharose HP column at a flow rate of 0.25 mL/min. The column was washed with the same buffer until no protein was detected. The bound lipase was eluted with Tris-HCl buffer, 100 mM NaCl and 0.33 mM CaCl_2_, pH 8.0. The active fractions were determined by SDS-PAGE gel electrophoresis and pooled, followed by concentration using Amicon Ultra-15 centrifugal filter (Millipore, Bedford, MA, USA). The concentrated solution was subjected to gel filtration in Sephadex G25 in XK16/20 column and pre-equilibrated with PBS buffer pH 7.4. The solution was run in the same buffer at a flow rate of 1 mL/min. The active fractions were collected and concentrated with an Amicon Ultra-15 centrifugal unit. The homogeneity of the partially purified protein was verified by SDS-PAGE and the protein was lyophilized and stored at −20 °C [11].

### 3.5. Standard Lipase Assay and Protein Concentration

The standard assay for the determination of lipase activity was carried out according to previous work [10]. The protein content was determined by the method of Bradford (1976). Amresco assay reagent and bovine serum albumin (BSA: Sigma-Aldrich, St. Louis, MO, USA) were used as the standard. The different concentrations of bovine serum albumin (BSA) were prepared from a stock solution of BSA (0.1 mg in 10 mL of dH_2_O) and a spectrophotometer (HITACHI U-3210) was used to monitor the protein content at 595 nm using preparations without BSA as blank [34].

### 3.6. Enantioselective Esterification of (R-S)-Ibuprofen with Oleyl Alcohol

The reaction consisted of lyophilized lipase powder (5 mg), (*R*,*S*)-ibuprofen (0.8252g, 4 mmol) and oleyl alcohol (1.872 mL, 6 mmol) and isooctane (unless otherwise stated) (8 mL) in screw-capped flasks (25 mL). The mixture was stirred at 200 rpm in a thermoconstant oil bath at 50 °C. Periodically, samples from each reaction mixture (100 μL) were collected and put in ice to stop the reaction. The mixtures were diluted appropriately for chiral HPLC analysis. Reaction samples (0.5 mL) were also withdrawn and mixed with hexane (2 mL).

#### Effect of Reaction Conditions

The esterification reactions were carried out in screw-capped flasks (25 mL) and were subjected to similar conditions of 50 °C, lyophilized lipase (5 mg), (*R*,*S*)-ibuprofen (0.8252 g, 4 mmol) and oleyl alcohol (1.872 mL, 6 mmol) and stirred at 200 rpm. Investigation on the effect of various solvents was carried out using solvents, DMSO, acetonitrile, ethyl acetate, hexane, isooctane, toluene, dodecane and *N*-tetradecane (8 mL). Assessment of the presence of the variable amount of desiccant was performed in mixtures containing molecular sieve contents of 0, 2 and 4 mg.

### 3.7. Analysis and Determination of Ibuprofen Esters

Analysis of the reaction mixture was performed on an Agilent 1200 HPLC equipped with a chiral phase column: (*R*,*R*)-Whelk-O1 chiral column (Regis Technologies, Morton Grove, IL, USA) and an ELSD detector for detection of all products. The eluent consisted of a mixture of hexane, isopropanol and acetic acid (98:2:0.5, *v*/*v*/*v*). The flow rate was kept at 0.75 mL/min and the column temperature at 25 °C. Sample injections were carried out by an autosampler. Samples (10 μL) were injected and the respective retention times of (*R*)-ibuprofen acid, (*S*)-ibuprofen acid, (*R*)-ibuprofen ester and (*S*)-ibuprofen ester were 9.2, 10.5, 5.4 and 5.7 min. The conversion, (*X*), of ibuprofen was determined by titration with NaOH (0.03 M). The conversion of ibuprofen ester was calculated using the following Equation 1;

(1) $$X\;(\%)=\frac{C_{\text{o}}-C_{i}}{C_{\text{o}}}\times 100$$

where *X* is the overall conversion, *C*_o_ the initial amount of racemic ibuprofen (mM) and *C*_i_ the amount of racemic ibuprofen at a particular reaction time (mM). The *E* values and e.e*_p_*% were calculated using Equations 2 and 3 as previously described in literature [35]. The values were an average of three measurements from two separate determinants. Meanwhile, [*R*]_ester_ and [*S*]_ester_ represents the concentration of the (*R*) and (S) enantiomers of ibuprofen esters, respectively.

(2) $${\text{ee}}_{\text{p}}(\%)=\frac{{\left[R\right]}_{\text{ester}}-{\left[S\right]}_{\text{ester}}}{{\left[R\right]}_{\text{ester}}+{\left[S\right]}_{\text{ester}}}\times 100$$

(3) $$E=\frac{\text{ln}[1-(X/100)(1+({\text{ee}}_{\text{p}}/100))]}{\text{ln}[1-(X/100)(1-({\text{ee}}_{\text{p}}/100))]}$$

## 4. Conclusions

Mutation at the oxyanion Q114 had a profound effect on enantioselectivity of reactions. Substitution with Met improved enantioselectivity, but Leu did not. From the synthetic point of view, solvent properties were found to influence the activity and selectivity of the lipase-catalyzed reactions. The *E*-values of Q114L, Q114M and T1 lipase correlated better with the log *p* of solvents, with the exception of hexane. Meanwhile, the low ester conversions were attributed to practical limitations set by the acidic pH of reaction that was far below the pH optimum of the lipase variants and incompatible use of desiccant. Extending the lipase range of pH tolerability towards lower pH values and using other desiccants as additives could improve the lipase-catalyzed esterification reaction.
